# Intravitreal Avastin as an adjunct in patients with proliferative diabetic retinopathy undergoing pars plana vitrectomy

**DOI:** 10.12669/pjms.292.3044

**Published:** 2013-04

**Authors:** Yawar Zaman, Aziz-ur Rehman, Abdul Fattah Memon

**Affiliations:** 1 Dr. Yawar Zaman, FRCS, Al-Ibrahim Eye Hospital, Isra Post Graduate Institute of Ophthalmology, Karachi, Pakistan.; 2 Dr.Aziz-ur-Rehman, FCPS, Al-Ibrahim Eye Hospital, Isra Post Graduate Institute of Ophthalmology, Karachi, Pakistan.; 3 Dr. Abdul Fattah Memon, FCPS, Al-Ibrahim Eye Hospital, Isra Post Graduate Institute of Ophthalmology, Karachi, Pakistan.

**Keywords:** Intravitreal bevacizumab, Proliferative diabetic retinopathy, Vascular endothelial growth factor, Diabetic vitrectomy

## Abstract

***Objective: ***Intravitreal Bevacizumab (Avastin, Genentech Inc., San Francisco, CA) (IVB) has been shown to cause regression of neovessels in proliferative diabetic retinopathy due to its anti-angiogenic effects. This study was performed to investigate the role of Avastin as an adjunct to the management of patients with proliferative diabetic retinopathy undergoing pars plana vitrectomy.

***Methodology: ***Fifty four eyes of 54 patients with proliferative diabetic retinopathy scheduled for surgery were included in the study. They were randomized to vitrectomy with preoperative IVB (group one) or standard vitrectomy (group 2). Group one underwent a single intravitreal injection of bevacizumab 1.25 mg /0.05ml one week prior to vitrectomy. Main outcome measures were best corrected visual acuity (BCVA) after surgery, post-operative complications.

***Results: ***Mean age of the patients was 52.07±5.54 years (range 39-67). At 6 months, 20 patients in group one had BCVA better than baseline as compared to 12 patients in group 2. In group one, only one patient had early post-operative vitreous hemorrhage, whereas 11 patients in group two had early vitreous hemorrhage.

***Conclusion: ***Preoperative IVB is helpful in improving BCVA post operatively, reducing the time of surgery, decreasing the incidence of intraoperative and postoperative bleeding and reducing the frequency of rubeosis and hyphaema.

## INTRODUCTION

The aim of vitrectomy in proliferative diabetic retinopathy is to re-establish visual acuity through removal of vitreous blood, removal of fibrovascular proliferation causing traction and to stabilize the neovascular process through panretinal endophotocoagulation of ischemic retina.

Intravitreal bevacizumab (IVB) has been shown to effectively reduce rubeosis and retinal neovascularization in proliferative diabetic retinopathy (PDR).^1^^,^^2^ Also, administration of IVB prior to diabetic vitrectomy may reduce intraoperative bleeding and post-operative complications in patients with tractional retinal detachment (TRD).^3^ Recurrent vitreous hemorrhage is a common indication for reoperation. Most of the hemorrhages occurs during the first 6 months but may occur years later.^4^

Bevacizumab (Avastin,) a full length humanized monoclonal antibody to vascular endothelial growth factor (VEGF), initially approved by the US Food and Drug Administration (FDA) for the treatment for metastatic colorectal cancer,^5^ has now been used in age-related macular degeneration and proliferative diabetic retinopathy(PDR).^2^^,^^6^ It has also been shown to clear the vitreous hemorrhage rapidly and induce regression of retinal neovascularization.^7^ This prospective study was conducted to investigate the effect of IVB prior to diabetic vitrectomy and on its postoperative course.

## METHODOLOGY

Fifty four eyes of 54 patients were included in the study, which was approved by the institutional review board. Study duration was one year i.e. from 1^st^ September 2010 to 31^st^ August 2011. The study was conducted at Al Ibrahim eye hospital, Karachi.

These patients were diagnosed with proliferative diabetic retinopathy (PDR) and were advised to undergo pars planavitrectomy (PPV). They were randomized into two categories. In group one, intravitreal bevacizumab (IVB) was injected 5 to 7 days prior to surgery (30 patients) and group two underwent standard PPV (24 patients). All treatment options and the off-label use of intravitreal bevacizumab were discussed with the patient; all patients provided written informed consent. The inclusion criteria were non clearing vitreous hemorrhage of at least one month, tractional retinal detachment (TRD) involving or threatening themacula, and pre-retinal subhyaloid bleeding covering the macula. Preoperative assessment included best-corrected visual acuity (BCVA), funduscopy with 90D lens and B-Scan ultrasonography in 9 patients was used as fundus was not visualized clinically. Two patients had visually significant cataract leading to unability to visualize the fundus thus B-scan ultrasonography was used in such patients. All patients were followed up postoperatively at day 1, day 7, day 14, and then monthly up to 6 months. The main outcome measures were improvement of BCVA after surgery, post-operative complications hyphema and rubeosis and frequency of vitreous hemorrhage. Early postoperative vitreous hemorrhage was taken as vitreous hemorrhage occurring within four weeks after surgery. All cases completed a minimum follow up of 6 months.

The patients in group 1 underwent a single injection of IVB one week prior to vitrectomy. After sterile preparation and draping, 1.25 mg/0.05 ml bevacizumab (Avastin, Genentech) was injected intravitreally in the operating theatre under topical anesthesia. Topical antibiotic (moxifloxacin) was started a day before the procedure and was continued for three days post injection. Best corrected visual acuity (BCVA) and ophthalmic evaluation were done on each visit pre and post operatively.

Standard 20 gauge 3-port PPV was performed using Alcon Accurus surgical system. Tractional membranes were removed using peeling, segmentation, delamination and en bloc dissection. Panretinal photocoagulation (PRP) was done at the end of surgery. Internal tamponade used was either air or silicone oil 5000cSt.

## RESULTS

Fifty four eyes of 54 patients were included in the study. Mean age of the patients was 52.07+5.54 years (range 39-67). There were 32 males and 22 females. Twenty four patients were included in group one while remaining 30 were included in group 2. Out of 54 eyes in 35 (64.8%) tamponade was not used while in rest of the 19 (35.2%) eyes, silicone oil was used. Post-operative BCVA is shown in Table-I. BCVA before the surgery was noted on proforma and it was compared with the BCVA post operatively, change in BCVA was observed as either improvement, deterioration or no change.

Patients in the group one had much better visual acuity than patients in group two. Most of the patients 18(75%) had their BCVA in the range of 6/60 or better compared to group 2 where most of the patients 14 (46%) had BCVA less than 6/60. In group 1, 20 (83%) patients had visual improvement while three (13%) had no change and only one (4%) had worsening of BCVA. On the other hand in group two, 12 (40%) patients had improvement in BCVA while 16 (53%) had no change and one (7%) had worsening of BCVA (Table-II). No significant difference was observed in the frequencies of postoperative rubeosis and hyphema among the groups. Table-III and IV shows the frequencies of rubeosis and hyphema among the two groups.

**Table-I:** Post op BCVA in both groups

	*6/6-6/618*	*6/24-6/6/60*	*FC*	*HM*
Group 1	3	18	2	1
Group 2	0	11	14	5

**Table-II:** Comparison of BCVA improvement among the groups

	*Group 1*	*Group 2*
improved BCVA	20	12
Same BCVA	3	16
Worse BCVA	1	2

**Table-III:** Frequencies of vitreous hemorrhage among the groups

	*No VIT HG*	*Early Vitreous HG*	*Late Vitreous HG*
Group 1	21	1	2
Group 2	10	11	9

**Table-IV:** Frequency of Hyphaema and rubeosis among groups

	*Rubeosis*		*Hyphaema*	
	No	Yes	No	Yes
Group 1	21	3	23	1
Group 2	23	7	28	2

In group one, only three (12.5%) patients had vitreous hemorrhage, two of them had it in later stage. In group two, 20 (60%) patients had vitreous hemorrhage. Out of these, 11 had it in early post-operative period. The difference in both groups was statistically significant with p value of less than 0.05, using independent t test.

## DISCUSSION

This prospective study was conducted to investigate the effect of IVB on diabetic vitrectomy. In our study, patients in the group one, who had IVB before PPV had much better post-operative visual acuity than patients in group two who had standard PPV. This is comparable to the study by El-Batarny where vision improved in 87% in IVT with PPV group and 80% in standard PPV group.^8^ Similarly in another study by Ahmadieh, BCVA was better in the IVB group at one month compared with the control group (P<0.004).^9^

The incidence of early postoperative vitreous hemorrhage was very low in group one where only three patients had vitreous hemorrhage. In the other group, 20 patients had vitreous hemorrhage, out of these 11 had it in early post-operative period. It has been shown in a number of studies that IVB may reduce the incidence of intraoperative and postoperative hemorrhage in diabetic vitrectomy.^3^^,^^10^^-^^12^

It is usually difficult to determine the source of early postoperative vitreous hemorrhage. Surgeons believe that dissected fibrovascular membranes are the source of bleeding which typically bleed within one week of surgery.^13^ In our Avastin-treated group, only three cases of postoperative bleeding were noticed.

Furthermore, IVB prior to surgery significantly reduced the duration of surgery. Easier dissection due to the absence of intraoperative bleeding and clear view seem to be the reasons for the reduction in the operating time.

Fewer cases in Avastin group had postoperative rubeosis or hyphaema. El-Batarny also reported a similar finding^8^ Retinal ischemia leads to an increased production of intravitreal VEGF, while inhibition of VEGF activity via IVB decreases VEGF levels and inhibits retinal and iris neovascularisation.^14^

In conclusion, intravitreal Bevacizumab reduces retinal neovascularization, thus resulting in better visual acuity postoperatively and reduction in intra and post operative complications when it is used preoperatively in pars planavitrectomy surgery in patients with proliferative diabetic retinopathy. More studies with adequate sample size are required to confirm this effect.

## Authors contribution

***YZ:*** Performed all surgeries.

***AR:*** Supervised the whole research project

***AFM:*** Study design and data analysis.
